# Income Trajectories and Subjective Well-Being: Linking Administrative Records and Survey Data

**DOI:** 10.3390/ijerph16234779

**Published:** 2019-11-28

**Authors:** Ina Schöllgen, Norbert Kersten, Uwe Rose

**Affiliations:** Federal Institute for Occupational Safety and Health (BAuA), Division 3 “Work and Health”, 10317 Berlin, Germany

**Keywords:** income, subjective well-being, life satisfaction, emotional well-being, change, trajectories, survey, administrative records, employees

## Abstract

An association between income and life satisfaction has been well documented, however, little is known of how income trajectories affect different facets of subjective well-being (SWB). The aim of this study was to examine how several aspects of income dynamics are related to life satisfaction and affect balance. Longitudinal information on income from administrative records covering 13 years (1999–2011) is linked to cross-sectional data on SWB collected in 2011/12 from the nationally representative Study on Mental Health at Work (S-MGA; *n* = 3364). Parameters from subject-specific regression analyses of income over time were used as indicators of income development in regressions over all participants, conducted separately for men and women. Associations between income and life satisfaction were stronger and more consistent than associations between income and affect balance. Major findings were that longer-term income change was more strongly related to SWB than current deviation from expected income. Higher stability in income development was associated with higher SWB. A higher share of income from benefits predicted lower life satisfaction and a more negative affect balance. Our results show the importance of examining income trajectories and taking into account source of income to gain a more differentiated view on the income-SWB association.

## 1. Introduction

Subjective well-being (SWB) is increasingly recognized as an important outcome in public health and has already been used to study socioeconomic inequalities in health (e.g., [1,2]). In particular, the association between income and SWB has been a flourishing area of study as the question whether money increases well-being is not only of academic interest but also of relevance for society and politics [3]. Prior studies have shown that there is robust evidence for an association between income and life satisfaction (for an overview see, e.g., [4]). Most of this research did not take into account that subjective well-being has two facets: an evaluative component, i.e., life satisfaction, and an emotional component, i.e., the frequency and intensity of positive emotions and (the absence of) negative emotions [5]. Moreover, information on income trajectories, i.e., short-term deviations and long-term developments, volatility (or stability) in income development and source of income, has not been fully explored regarding relevance for SWB in the same study. The main contribution of this study is to examine the link between different income characteristics over time and life satisfaction as well as affect balance as indicators of SWB. Gender differences in the income-SWB association will be taken into account. In the following, more details on the scientific background will be provided, before presenting the objectives and hypotheses guiding our research.

### 1.1. Income and Different Facets of Subjective Well-Being

As Kahneman and Deaton [6] have shown, the two facets of subjective well-being, i.e., evaluative and emotional, are differentially related to income status at one point in time, and there is some evidence that long-term changes of *national* income are more strongly related to evaluative judgements of life compared to emotional well-being [7]. It is an open question whether associations of life satisfaction and affective well-being, respectively, with *individual* income developments over time also differ. Of particular importance in this context, the time frame used to assess life satisfaction and emotional well-being usually differs: the occurrence of positive and negative emotions is evaluated over a specific period of time, whereas life satisfaction is rated without providing a specific time frame [8]. This might affect associations with income dynamics. In particular, if emotional well-being is evaluated over a shorter period of time like weeks or months, this might be more strongly affected by more recent developments in income, whereas the overall evaluation of ones’ life might rather be affected by long-term trends in income.

Some evidence for this assumption is provided in a study by Oshio and colleagues [9] linking career wage records covering a period of over 30 years to current life satisfaction, distress, and self-rated health in a sample of male employees in Japan. In this study, aggregate measures and difference scores have been used to calculate a range of income indicators, rather than estimating income slopes. The findings showed that life satisfaction was related to differences between current and maximum income as well as current and lifetime average income (i.e., a proxy for long-term income trends), but was unrelated to differences between current income and income in the previous year, reflecting short-term developments. Results were different for psychological distress, reflecting negative emotions and experiences: this indicator was sensitive to short-term changes in income (current—previous year), but was not related to differences reflecting a longer time span, i.e., between current and lifetime average income. Overall, these findings suggest that different facets of subjective well-being (and ill-being) might be differentially related to income dynamics. In the following, we will take a closer look at (temporal) income characteristics and well-being. 

### 1.2. Income Characteristics and Well-Being

There is a vast literature examining the association between income status and subjective well-being at one point in time, and a growing number of studies based on longitudinal survey data investigating whether an increase or decrease in income between adjacent time points is reflected in an increase or decrease in life satisfaction [10,11,12]. Although this approach provides some insights into the effects of income dynamics, it does not allow for distinguishing between long-term trends and short-term deviations in income and their respective effects on different facets of SWB.

Another area of growing research and public health interest concerns the effect of income volatility on health and well-being [13]. Higher volatility or instability in income is assumed to reflect unpredictability and financial insecurity, and hence to be negatively related to (mental) health [14,15]. Previous studies examining the link between income volatility and mental health focused on depressive symptoms rather than subjective well-being indicators and focused on instability in income rather than fully exploring the health relevance of different income characteristics over time.

Apart from temporal characteristics, source of income might also matter for subjective well-being [4]. Although data collection often comprises income from different sources, those are usually not distinguished in studies on income and well-being. An exception is a study by Ahn and colleagues [16] who found that labour income was more strongly related to financial satisfaction (as a domain-specific aspect of SWB) compared to non-labour income. The procedural utility hypothesis, i.e., “that people value not only actual outcomes, i.e., the what, but also the conditions and processes which lead to these outcomes, i.e., the how” ([17], p. 2), may explain these findings. Looking at income characteristics over time and using a broad outcome measure of psychological well-being, a study by Kaplan and colleagues [18] suggests that source of income affects well-being beyond the amount of income and change in income. More specifically, their results show that more periods of receipt of benefit income were associated with lower well-being. Taken together, theoretical assumptions and first empirical results suggest to take into account source of income when examining income-SWB associations.

### 1.3. Income and Well-Being: Gender Differences

Taking a closer look at the income-SWB association also entails taking into account interindividual differences that might moderate this effect. As stated by Diener and Biswas-Diener “the effects of money on SWB differ depending on ones’ life circumstances, roles, and values” ([3], p. 128). In line with this statement, the authors present evidence for gender differences in the association between income and well-being, which might be explained by different societal roles and expectations for men and women, with the male gender role emphasizing economic success. A study examining associations between income and subjective financial well-being, i.e., a domain-specific well-being indicator, found that only in men, but not in women, income was related to subjective financial well-being [19]. Taken together, these studies suggest that, overall, the association between income and SWB might be stronger in men compared to women. However, it is an open question how gender differences play out with regard to different income characteristics over time.

### 1.4. Aims and Hypotheses

The aim of the present study was to examine how several aspects of income dynamics are related to two facets of subjective well-being, i.e., life satisfaction and affect balance. Our first objective was to examine the effects of longer-term income change (i.e., over 13 years) and current income deviation (i.e., the deviation from expected income in the current year) on life satisfaction and affect balance. Effects of stability in income development on evaluative and emotional facets of SWB (Objective 2) and the role of source of income for life satisfaction and affect balance (Objective 3) will also be investigated. As outlined in more detail above, stability in income development and source of income constitute potentially important but rarely investigated predictors of well-being.

We hypothesize that life satisfaction is more strongly linked to longer-term income change than current income deviation, whereas affect balance is more strongly affected by current income deviation than longer-term income change (H1). Higher stability reflects a nearly monotonic income development, i.e., less ups and downs in income over time. As income volatility is assumed to reflect unpredictability, and hence to be negatively related to well-being [15], we expect higher stability in income development to be associated with higher life satisfaction and a more positive affect balance (H2). Regarding source of income, we expect that a higher share of income from benefits predicts lower life satisfaction and a more negative affect balance (H3).

Gender differences in the associations between longer-term income change, current income deviation, stability in income development and source of income with life satisfaction and affect balance will be examined in an exploratory fashion. Our results offer a more differentiated view on the association between income and subjective well-being.

## 2. Materials and Methods

### 2.1. Sample

Data come from the Study on Mental Health at Work (S-MGA). The S-MGA sample is based on the Integrated Employment Biographies (IEB), which combine data from several sources of the German Federal Employment Agency [20]. The IEB covers employees in Germany subject to social security contributions, which constitutes more than 80% of all employees in Germany [21] on the reference of sampling (31 December 2010). Civil servants, self-employed individuals and freelancers are not included by this definition, however, the defined population and hence the sample includes employees with full-time and part-time jobs including marginal part-time employment [22]. A two-stage cluster sampling was applied for S-MGA with a random selection of 206 municipalities at the first stage and a random selection of individuals within municipalities at the second stage. Individuals who were born between 1951 and 1980 were included. All subjects gave their informed consent for inclusion before they participated in the study. The study was conducted in accordance with the Declaration of Helsinki, and the protocol was approved by the Ethics Committee of the Federal Institute for Occupational Safety and Health (BAuA; Project F2384).

In 2011/2012, data collection took place via computer-assisted personal interviews and questionnaires covering a broad range of topics on work and (mental) health, resulting in a sample of 4511 respondents (with a response rate of 35.7% according to the standards of the American Association for Public Opinion Research (AAPOR [23]), similar to other German surveys; e.g., [24,25]). Analyses reported by Rose and colleagues [22] show that there are only minor deviations between the population, gross sample and the sample of respondents regarding socio-demographic characteristics, providing no indication for a sampling bias. For example, the difference in full-time employment was −1.1%, with 68.4% of employees in the population being employed full-time whereas 67.3% of the sample of respondents were employed full-time (the difference in marginal part-time employment was −1.3%, i.e., 12% of the population and 10.7% of the sample of respondents being marginally employed, [22]). Respondents were asked to link their interview and questionnaire data with their employment histories from the IEB, containing information on employment status and income on a daily basis from 1999 to 2011 [20]. 3364 participants (74.6%; 1657 men and 1707 women) agreed to this. Selectivity analyses show that they do not differ from the full sample regarding gender, age, vocational training, employment status, and cohabiting with a partner (see Supplementary Table S1). After excluding data sets with missing values, the remaining sample size for the analyses on life satisfaction is *n* = 3152, 1566 men and 1586 women, and for the analyses on affect balance the sample size is 2903 (1446 men and 1457 women).

### 2.2. Measures

#### 2.2.1. Income

Administrative records on income from the German Federal Employment Agency were used to compute income per year from 1999 to 2011, yielding up to 13 data points for each subject. To reduce the amount of data per subject, subject-specific regression analyses of income over time were carried out and the regression parameters were used as indicators of income development. For the analyses, several indicators were used: expected income in 1999 (i.e., the intercept), mean change in income (i.e., the slope), current income deviation (i.e., the residual in 2011), stability in income development (measure of determination, i.e., the proportion of explained variance for the linear trend standardized by within subject variance, with a range from 0 to 1) and source of income (share of income from benefits, relative to total income 1999–2011, with a range from 0 to 1). The measure of determination was used as an indicator of income stability, i.e., the invariance and linearity, over time, since it can be assumed to be a measure of planning certainty with regard to income development.

#### 2.2.2. Subjective Well-Being

Life satisfaction was assessed with the German version of the Satisfaction with Life Scale [26]. Five items were rated on a seven-point scale (1 = do not agree at all, 7 = fully agree). An item example is “I am satisfied with my life”. A summary score is used to calculate life satisfaction, ranging from 7 to 35. Scale reliability calculated as Cronbach’s alpha is 0.834 for men and 0.863 for women. Positive and negative emotions were measured with the Scale of Positive and Negative Experiences [27]. Participants were asked how often (1 = very rarely or never to 5 = very often or always) they experienced six positive emotions and states (positive, good, pleasant, happy, joyful, contented) and six negative emotions and states (negative, bad, unpleasant, sad, afraid, angry) during the last four weeks. The reliability of the scale of positive emotions, calculated as Cronbach’s alpha, is 0.859 for men and 0.874 for woman. The corresponding numerical values for the scale of negative emotions are 0.829 and 0.828, calculated as Cronbach’s alpha. As the German version of the Scale of Positive and Negative Experiences which was validated by [28] was not published yet in 2011, an own translation was used which slightly differs for two out of 12 adjectives used (see Table S2 in the Supplementary Materials). The factorial structure of the scale used in the present study was examined using confirmatory factor analysis, conducted with Mplus version 8.1 (Muthén & Muthén, Los Angeles, CA, USA). The results, presented in more detail in the Supplementary Materials (Table S3) provided support for two factors, i.e., positive emotions and negative emotions, which were highly correlated (*r* = −0.71; fit for the 2-factor model with two correlations between error terms permitted: χ^2^(51) = 315.981, comparative fit index/CFI = 0.975, root mean square error of approximation/RMSEA = 0.042, standardized root mean squared residual/SRMR = 0.025; model depicted in Figure S1; see also [29]). Moreover, the same factor structure could be found in men and women (fit for model with equal factor loadings: χ^2^(122) = 555.341, CFI = 0.960, RMSEA = 0.049, SRMR = 0.042; see Table S3). Hence, following the suggestion by Diener and colleagues [27], we created a score of affect balance by summing up the positive emotions and the negative emotions separately, with each scale ranging from 6 to 30, and then subtracting the negative score from the positive score, with affect balance scores ranging from -24 to +24.

#### 2.2.3. Confounders

A number of variables were tested to see whether they have an independent influence on well-being and whether they are associated with income. As potential confounders, age (in years), level of vocational training (1 = no/other, 2 = apprenticeship, 3 = vocational education including additional qualification or a university degree) and cohabiting with a partner (0 = yes, 1 = no) resulted. We computed age in 1999; information on vocational training and cohabitation were assessed in the survey.

### 2.3. Analyses

Analyses were completed with SPSS version 25 (IBM, Armonk, NY, USA). We first computed linear regressions for each participant separately to obtain parameters reflecting individual trends (e.g., the β-coefficients and the measure of determination). These subject-specific parameters were entered as variables in regressions over all participants, conducted separately for men and women. Analyses were stratified by gender to explore potential differences in the association between income dynamics and SWB. Normal probability plots were used to check whether residuals are normally distributed. The normal probability plots were approximately linear, supporting this assumption [30]. 

A series of models was estimated for each outcome, i.e., life satisfaction and affect balance. We first computed models containing all confounders (age, vocational training, cohabitation) and one income indicator each, i.e., expected income in 1999 (Model 1), mean change in income (Model 2), current income deviation (Model 3), stability in income development (Model 4) and share of income from benefits (Model 5). Then, three models were estimated containing all covariates as well as several income indicators: Model 6 included expected income in 1999, mean change in income, and share of income from benefits, whereas Model 7 included mean change in income and current income deviation and Model 8 contained stability in income development and share of income from benefits. Due to issues of multicollinearity, it was not possible to estimate parameters for all income indicators in one model. As our primary interest was to examine links between individual income trajectories and subjective well-being rather than investigating links between relative income position and well-being, we did not use a logarithmic transformation for income.

## 3. Results

Descriptive statistics, stratified by gender, are reported in Table 1. There were no gender differences in age. Regarding highest degree of vocational training, more men than women had a university degree, whereas the proportion of the sample that completed an apprenticeship was higher in women compared to men. A higher proportion of men compared to women was cohabiting with a partner. Life satisfaction was higher in women, but there was no gender difference in affect balance. Regarding income characteristics, expected income in 1999 was higher in men, and there was a stronger increase in income over time, on average, in men compared to women. Current income deviation differed by gender, with women tending to have a positive income deviation, i.e., a higher income in 2011 than would be expected. Stability in income development was higher among men, whereas there was no gender difference in share of income from benefits. The correlation between life satisfaction and affect balance, was *r* = 0.456 in male subjects and *r* = 0.494 in female subjects. The full correlation tables can be found in the Supplementary Materials (Tables S4 and S5).

### 3.1. Income and Life Satisfaction

Regression results for income dynamics and life satisfaction are reported in Table 2. Overall, it can be seen that income is more strongly related to life satisfaction in men than in women.

We first report results from models containing all covariates and one income indicator each. Higher expected income in 1999 (i.e., the intercept) is associated with higher life satisfaction in 2011/12 in men (*b* = 0.041, *p* < 0.001; Model 1), but only weakly associated with life satisfaction in women (*b* = 0.021, *p* < 0.05). Regarding mean change in income from 1999 to 2011 (i.e., the slope), an increase in income predicts higher life satisfaction in men (*b* = 0.548, *p* < 0.001) and women (*b* = 0.307, *p* < 0.01; Model 2). On the other hand, falling income developments also occur (i.e., a negative slope), which are associated with lower life satisfaction. Coefficients reflect the effect of an income difference or change, respectively, of 1000€ on life satisfaction. Current income deviation (i.e., the residual in 2011) was only related to life satisfaction in men (*b* = −0.078, *p* < 0.001), but not in women (*b* = −0.005, *p* = 0.829; Model 3). For both men and women, higher stability in income development (i.e., higher values for the measure of determination) is associated with higher life satisfaction (men: *b* = 2.082, *p* < 0.001; women: *b* = 1.159, *p* < 0.01; Model 4, Table 2). Varying and unstable income (i.e., lower values for the measure of determination), on the other hand, results in lower life satisfaction. Regarding source of income, those with a higher share of income from benefits (relative to total income 1999–2011) report lower life satisfaction in 2011/12 (men: *b* = −15.026, *p* < 0.001; women: *b* = −9.892, *p* < 0.001; Model 5). Hence, the difference in life satisfaction for men gaining all of their income from benefits compared to men gaining none of their income from benefits in the 13 years prior to well-being assessment amounts to 15 points on a scale ranging from 7 to 35; for women, this difference amounts to almost 10 points. As shown in Table 1, most of the participants gained no or a small amount of income from benefits. However, 532 men and 542 women gained some of their income from benefits, with the maximum share of income from benefits (relative to total income 1999–2011) amounting to 77.8% in men (75 percentile: 9.5%, 90 percentile: 17.8%) and 86.6% in women (75 percentile: 9.4%, 90 percentile: 21.3%).

As can be seen in Table 2 (Model 6), associations of expected income in 1999, mean change in income, and source of income with life satisfaction are still significant when mutually adjusted for each other. In men, current income deviation is still significantly associated with life satisfaction if mean change in income is controlled for (Model 7, Table 2). The association is negative, which means that a positive income deviation (i.e., a higher income in 2011 than would be expected) is associated with lower life satisfaction, whereas a negative income deviation (i.e., a lower income in 2011 than would be expected) is associated with higher life satisfaction. Further analyses (see Supplementary Table S6) show that more than half of those participants having a long-term increase or no change in income show a negative income deviation, i.e., a lower income in 2011 than expected (men: 55%, women: 52%). On the other hand, more than two third of participants having a long-term decrease in income show a positive (or no) income deviation in 2011 (men: 68%, women: 75%; Table S6). As can be seen in Table 2, the association of mean change in income with life satisfaction is stronger than the association of current income deviation with life satisfaction (Model 7). Results from Model 8 show that, for men, stability in income development and source of income are associated with life satisfaction independent of each other, whereas for women, the effect of income stability becomes non-significant when source of income is controlled for.

In men and women, cohabiting with a partner was associated with higher life satisfaction in multivariate analyses. The association of higher age with lower life satisfaction was significant in most multivariate models in men and women. Regarding vocational training, compared to men and women having completed an apprenticeship, those with no or other vocational training report lower life satisfaction—this effect was significant in most multivariate models. The positive effect of a university degree on life satisfaction in men lost its significance in models containing mean change in income. Concerning explained variance in life satisfaction, R² of the multivariate models ranged from 0.08–0.11 in women and from 0.09–0.14 in men.

### 3.2. Income and Affect Balance

Regression results for income dynamics and affect balance are reported in Table 3. Comparing Table 2 and Table 3, overall, it can be seen that income is less strongly related to affect balance than to life satisfaction in men and in women.

Again, we first report results from models containing all covariates and one income indicator each. Expected income in 1999 (i.e., the intercept) is not associated with affect balance in 2011/12, neither in men (*b* = 0.011, *p* = 0.372) nor in women (*b* = 0.010, *p* = 0.758; Model 1). Regarding mean change in income (i.e., the slope), an increase in income predicts a more positive affect balance in men (*b* = 0.367, *p* < 0.01), but not in women (*b* = 0.179, *p* = 0.228; Model 2). Current income deviation (i.e., the residual in 2011) was only related to affect balance in men (*b* = −0.073, *p* < 0.01), but not in women (*b* = 0.015, *p* = 0.634; Model 3). Higher stability in income development (i.e., a higher value on the measure of determination) is associated with a more positive affect balance in men only (men: *b* = 1.403, *p* < 0.05; women: *b* = 0.871, *p* = 0.124; Model 4). Regarding source of income, those with a higher share of income from benefits (relative to total income 1999–2011) report a more negative affect balance in 2011/12 (men: *b* = −7.392, *p* < 0.01; women: *b* = −6.735, *p* < 0.01; Model 5, Table 3). This means that the difference in affect balance for men gaining all of their income from benefits compared to men gaining none of their income from benefits in the 13 years prior to well-being assessment amounts to more than 7 points on a scale ranging from −24 to +24; for women, this difference amounts to almost 7 points.

As can be seen in Table 3, when expected income in 1999, mean change in income, and source of income are mutually adjusted for each other, only income change retains its significant association with affect balance in men (Model 6). Source of income retains its significant association with affect balance above and beyond stability of income, however (Model 8, Table 3). In men, current income deviation is still significantly associated with affect balance if mean change in income is controlled for (Model 7). The association is negative, which means that a positive income deviation (i.e., a higher income in 2011 than would be expected) is associated with a more negative affect balance. As can be seen in Table 3, the association of mean change in income with affect balance is stronger than the association of current income deviation with affect balance (Model 7).

In men and women, cohabiting with a partner was associated with a more positive affect balance in multivariate analyses. The association of higher age with a more negative affect balance was significant in most multivariate models in men and women. Vocational training did not have an effect on affect balance in men, however, women with a university degree had a more positive affect balance compared to those with a completed apprenticeship—the latter effect was significant in most multivariate models. Concerning explained variance in affect balance, R² of the multivariate models ranged from 0.02–0.03 in women and from 0.03–0.04 in men.

## 4. Discussion

The present study used administrative records on income over up to 13 years linked to survey data of a nationally representative sample of employees to investigate associations between income trajectories and life satisfaction as well as affect balance as an indicator of emotional well-being. Our analyses showed that, overall, associations with income were stronger for life satisfaction, i.e., the evaluative component of well-being, than for affect balance as an indicator of emotional well-being. Looking at the income trajectories in more detail, it was shown that longer-term income change (i.e., over 13 years) was associated with life satisfaction in men and women, whereas associations with affect balance were only apparent in men. Unexpectedly, current income deviation, i.e., the deviation from expected income in the last year, had a small negative effect on life satisfaction and affect balance in men. As hypothesized, higher income stability, reflecting a nearly monotonic income development, i.e., less ups and downs in income over time, was related to higher life satisfaction in men and women as well as a more positive affect balance in men above and beyond covariates. However, when source of income was taken into account, the effects of income stability largely disappeared. As expected, a higher share of income from benefits predicted lower life satisfaction and a more negative affect balance in both men and women. Findings will be discussed in more detail in the following.

### 4.1. Income Trajectories: What Matters for Subjective Well-Being?

A central finding of our study is that source of income, i.e., the share of income from benefits, was the only predictor related to life satisfaction as well as affect balance in both men and women. Our results extend the findings from Kaplan and colleagues [18] who have shown that more periods of receipt of benefit income were associated with poorer well-being. In this prior study, it was not possible to determine how much income is received from profit income or from benefit income, which might have resulted in an underestimation of the impact of source of income (see [18]). An advantage of the present study was that administrative records on income were available, allowing for calculation of the amount of profit income and benefit income and hence for determining the share of income from benefits over a period of 13 years. In our sample, more than one third of participants derived at least part of their income from benefits at some point in time, with a sizeable proportion having a share of income from benefits of more than 10%, showing the relevance of this source of income. Taken together, our results and the findings from Kaplan and colleagues [18] as well as Ahn and colleagues [16] show that well-being is not only be affected by the *amount* of income, as many studies have shown, but that *source* of income also plays an important role. It is a major contribution of our study that we included source of income as a rarely examined but important predictor of subjective well-being. Future studies should look at potential mechanisms behind this effect, including economic security perception [31]. Further exploring the role of source of income for SWB is of not only scientific interest, but these results might also inform labour market policies [16].

Comparing the effects of longer-term income change and current income deviation, our results suggest that long-term trends more strongly relate to subjective well-being, except from affect balance in women, which was not related to either long-term trends or short-term deviations. According to Benzeval and Judge [32], longer-term increases or decreases in income, respectively, might better reflect the material resources available to an individual compared to income measured at one point in time. Our results suggest that, even if current income deviates from the expected income, this might be less consequential for subjective well-being than long-term income trends. For example, an increase in income over 13 years provides opportunities for asset formation, which might buffer the effect of a negative income deviation in 2011 on well-being assessed in the same year. Besides, additional analyses (reported in Section 3.1) suggested that unexpected income deviations occurred frequently, i.e., a negative income deviation in 2011 despite long-term increases, a positive or no income deviation in 2011 despite long-term decreases (see Supplementary Table S6). The small negative effect of current income deviation on well-being in men, which was unexpected, might hence rather reflect the long-term trend working in opposite direction. 

The small effect of current deviation in income, limited to men, should not lead to the conclusion that ups and downs in income over time do not play a role for well-being. Our results suggest that stability in income development over 13 years, operationalized with the measure of determination, is associated with subjective well-being, except from affect balance in women. As multivariate models (i.e., Model 8 in Table 2 and Table 3) suggest, this effect might be partly explained by lower (or higher, respectively) stability reflecting a higher (lower) share of income from benefits.

Overall, referring to the hypotheses (see Section 1.4), the results of our study provide partial support for H1. As expected, life satisfaction was more strongly linked to longer-term income change than current income deviation. The assumption that affect balance would be more strongly affected by current income deviation than longer-term income change was not supported. Support for H2 is mixed. Higher stability in income development was associated with higher life satisfaction in men and women as well as a more positive affect balance in men, but only the association with life satisfaction in men retained its significance when source of income was taken into account. Hypothesis 3, stating that a higher share of income from benefits predicts lower life satisfaction and a more negative affect balance, was supported by our results.

Gender differences in the associations were examined in an exploratory fashion as theoretical assumptions and results of prior studies indicated that, overall, income is more strongly related to SWB in men. Extending this research, the current study provides a more detailed picture concerning the role of several income characteristics for life satisfaction and affect balance in men and women. Comparing the regression coefficients and outcome variance explained, income characteristics seem to be more strongly related to life satisfaction in men compared to women. The picture is less clear for affect balance as most income characteristics explained very little variance in emotional well-being in both men and women. Source of income was associated with life satisfaction and affect balance in both men and women. Our results suggest that gender might not uniformly shape associations between income characteristics and SWB facets.

In line with previous studies, our results show that income characteristics only explain a small amount of variance in SWB, particularly in emotional well-being. Other predictors of SWB, as shown by previous reviews and meta-analyses, are personality traits [33], which are less amenable to intervention and hence difficult to address from a public health perspective, however. Furthermore, social relationships and social support have been shown to be important for subjective well-being [34] as well as physical health, particularly at higher ages [35]. For employees, psychosocial job characteristics have also been shown to be related to well-being, particularly to domain-specific indicators such as job satisfaction [36]. Concerning broader well-being indicators like life satisfaction, direct effects of job characteristics are rather small [37]. In general, to explain a multi-determined construct such as life satisfaction or affect balance, a broad range of predictors has to be considered. The present study, however, focused on income trajectories and evaluative as well as emotional aspects of well-being to offer a more differentiated view on the income-SWB association. 

### 4.2. Strengths and Weaknesses of the Current Study

A strength of the present study is that we included both facets of subjective well-being, i.e., evaluative (life satisfaction) as well as emotional (i.e., affect balance), using validated measures. Overall, our results support this distinction. The correlation between life satisfaction and affect balance was medium-high but not in a range suggesting that these are identical constructs. Besides, associations with income differed, with stronger and more consistent effects for life satisfaction than affect balance (see also [7]). However, our results suggest that source of income, in particular, is not only related to a person’s overall evaluation of his or her life but also to the experience of positive and negative emotions. Hence, both facets of subjective well-being should be included in future studies on income and well-being.

Reverse causality is a concern, as we did not have measures of subjective well-being in 1999, i.e., when income data were first provided. It could thus be argued that poorer income development is the result of lower subjective well-being. Indeed, results of studies investigating both directions of influence suggest that not only does income affect future subjective well-being, but that higher well-being also predicts higher (self-reported) earnings in the future (e.g., [38]). The present study provided a more detailed picture of the income characteristics that matter for SWB, but it cannot be inferred from our results that effects between income and SWB are unidirectional. 

An important advantage of the present study is that we used administrative records on income. Using administrative records addresses two problems frequently reported in studies on income and well-being: missing income data and problems with data quality [6]. Moreover, same-source bias is avoided. This is of particular importance for investigating associations between income and well-being as it has been shown that people who are more satisfied with their wage tend to overestimate their true wage when being asked to report their income, whereas those who are less satisfied underestimate their income [39]. Despite the advantages of this approach, administrative records have hardly been used for investigating associations between income trajectories and subjective well-being. It needs to be taken into account, however, that the administrative records used in the present study provide information on individual income rather than household income. For women, individual income might be a more inaccurate approximation of the financial situation overall compared to men [40]. The focus of our analyses was on income *trajectories* rather than income *differences*, however, and we stratified the analyses by gender to account for differential associations.

The administrative records provide information on income from labour and income from benefits, however, income from other sources such as income from capital is not included. Hence, the current study does not provide a full picture of individuals’ financial status. For example, it could be assumed that the effects of current income deviations on SWB are moderated by the amount of financial assets, i.e., the relevance of current income deviations might be most apparent for those with little or no assets. This could not be investigated in the current study, but might be an interesting avenue for future research.

Given our sample of employees that are subject to social security contributions, our results cannot be generalized to other groups of employees, such as civil servants, self-employed individuals and freelancers. Concerning generalizability to other countries, it needs to be taken into account that there are several determinants on the macro-level such as the welfare state type, the development of the social welfare legislation, and the flexibility of the labor market, which might impact the individual employment history and hence can modify associations found in the current study on German employees. It would be far beyond the scope of the study to quantify these modifying effects without any data from other countries at hand.

## 5. Conclusions

The present study investigated associations between register-based income trajectories and subjective well-being, i.e., life satisfaction as well as affect balance, in a nationally representative sample of employees in Germany, separately for men and women. Overall, income characteristics were more strongly related to life satisfaction in men compared to women. Three major findings emerged: long-term income change shows stronger associations with subjective well-being than current income deviation; source of income matters for subjective well-being; and, relatedly, (in)stability in income development is associated with well-being. In particular, stability in income development is important for life satisfaction. Supporting benefit is an important option in this context, but none that creates satisfaction. Our results show the importance of taking a differentiated look at the association between income and subjective well-being by examining income trajectories, including source of income, and distinguishing between different facets of subjective well-being.

## Figures and Tables

**Table 1:** Descriptive statistics for study variables for men (*n* = 1657) and women (*n* = 1707).

	Men		Women	
	Mean	SD	Mean	SD
Age in 1999	33.85	8.16	34.05	7.97
Vocational training				
University degree (%)	37.4		34.0	
Apprenticeship (%)	56.7		60.1	
No/other (%)	5.9		5.9	
Cohabiting partner in 2011/12 (%)	80.4		76.0	
Life satisfaction in 2011/12 ^a^	25.46	4.75	26.02	4.99
Affect Balance in 2011/12 ^b^	7.34	6.72	7.16	6.86
Expected income in 1999 (1000€)	25.72	16.27	14.87	13.34
Mean change in income (1000€)	1.08	1.42	0.73	1.26
Current income deviation (1000€)	−0.05	6.20	0.39	5.56
Stability in income development (0 to 1)	0.54	0.32	0.49	0.32
Share of income from benefits (0 to 1)				
- all individuals	0.02	0.07	0.03	0.08
- only individuals with benefits	0.07	0.10	0.09	0.13

^a^ for life satisfaction, *n* = 1570 (men) and *n* = 1587 (women). ^b^ for affect balance, *n* = 1450 (men) and *n* = 1458 (women).

**Table 2:** Income and life satisfaction: Associations in men (*n* = 1566) and women (*n* = 1586).

Model	Men			Women		
	b	SE	β	b	SE	β
1. Expected income in 1999 (1000€)	0.041 ***	0.008	0.140	0.021 *	0.009	0.058
2. Mean change in income (1000€)	0.548 ***	0.094	0.163	0.307 **	0.100	0.078
3. Current income deviation (1000€)	−0.078 ***	0.020	−0.095	−0.005	0.024	−0.005
4. Stability in income development (0 to 1)	2.082 ***	0.373	0.139	1.159 **	0.380	0.073
5. Share of income from benefits (0 to 1)	−15.026 ***	1.962	−0.187	−9.892 ***	1.473	−0.160
6. Expected income in 1999	0.049 ***	0.009	0.167	0.022 *	0.010	0.059
Mean change in income	0.650 ***	0.102	0.193	0.292 **	0.109	0.074
Share of income from benefits	−9.128 ***	2.107	−0.114	−8.547 ***	1.543	−0.138
7. Mean change in income	0.483 ***	0.000	0.144	0.334 **	0.000	0.085
Current income deviation	−0.049 *	0.000	−0.060	0.021	0.000	0.021
8. Stability in income development	1.488 ***	0.379	0.100	0.719	0.382	0.046
Share of income from benefits	−13.133 ***	2.012	−0.163	−9.375 ***	1.498	−0.151

Note: all models adjusted for covariates age in 1999, level of vocational training and cohabiting with a partner; b = unstandardized coefficient, β = standardized coefficient; * *p* < 0.05, ** *p* < 0.01, *** *p* < 0.001.

**Table 3:** Income and affect balance: Associations in men (*n* = 1446) and women (*n* = 1457).

Model	Men			Women		
	b	SE	β	b	SE	β
1. Expected income in 1999 (1000€)	0.011	0.012	0.026	0.010	0.014	0.021
2. Mean change in income (1000€)	0.367 **	0.141	0.079	0.179	0.148	0.033
3. Current income deviation (1000€)	−0.073 **	0.028	−0.068	0.015	0.032	0.012
4. Stability in income development (0 to 1)	1.403 *	0.566	0.067	0.871	0.566	0.040
5. Share of income from benefits (0 to 1)	−7.392 **	2.702	−0.072	−6.735 **	2.154	−0.081
6. Expected income in 1999	0.014	0.014	0.035	0.008	0.015	0.016
Mean change in income	0.368 *	0.157	0.079	0.137	0.163	0.025
Share of income from benefits	−5.053	2.981	−0.050	−6.174 **	2.260	−0.074
7. Mean change in income	0.298 *	0.000	0.064	0.218	0.000	0.040
Current income deviation	−0.060*	0.000	−0.055	0.029	0.000	0.024
8. Stability in income development	1.088	0.584	0.052	0.570	0.574	0.026
Share of income from benefits	−6.115 *	2.785	−0.060	−6.341 **	2.190	−0.076

Note: all models adjusted for covariates age in 1999, level of vocational training and cohabiting with a partner; b = unstandardized coefficient, β = standardized coefficient; * *p* < 0.05, ** *p* < 0.01, *** *p* < 0.001.

## Supplementary Materials

The following are available online at https://www.mdpi.com/1660-4601/16/23/4779/s1, Table S1: Selectivity analyses comparing the full sample (*n* = 4511) and the sample providing administrative records (*n* = 3364). Table S2: Items of the Scale of Positive and Negative Experiences (SPANE). Table S3: Results of confirmatory factor analysis (CFA) of the Scale of Positive and Negative Experiences. Table S4: Correlations between study variables in men. Table S5: Correlations between study variables in women. Table S6: Current income deviation (residual 2011) by mean change in income. Figure S1: Model 3 of CFA of the Scale of Positive and Negative Experiences (SPANE).
