# Supplementary material for: Income Trajectories and Subjective Well-Being: Linking Administrative Records and Survey Data
Source: Int J Environ Res Public Health. 2019 Nov 28;16(23):4779. doi: 10.3390/ijerph16234779 (PMC6926602; doi:10.3390/ijerph16234779)
Supplement: Supplementary file 1 [file ijerph-16-04779-s001.pdf]

Table S1. Selectivity analyses comparing the full sample (*N* = 4,511) and the sample providing administrative records (*n* = 3,364)

	**Sample with administrative records (*n* = 3,364)**	**Full sample (*N* = 4,511)**	**Difference**
Gender: female (%)	50.7	50.5	0.2
Age in 1999			
< 30 years (%)	33.3	32.2	1.1
30 – 39 years (%)	39.5	39.8	-0.3
40 – 49 years (%)	27.3	28.0	-0.7
Vocational training			
University degree (%)	35.7	35.2	0.5
Apprenticeship (%)	58.4	58.5	-0.1
No/other (%)	5.9	6.3	-0.4
Cohabiting partner in 2011/12 (%)	78.2	78.4	-0.2
Employment status in 2011/12			
Employed (%)	93.2	93.1	0.1
Not employed (%)	6.8	6.9	-0.1

Table S2. Items of the Scale of Positive and Negative Experiences (SPANE).

**English (Diener et al., 2009)**	**German (Rahm et al., 2017)**	**German (Schöllgen et al.)**
Positive	Positiv	Positiv
Good	Gut	Gut
Pleasant	Angenehm	Angenehm
Happy	Glücklich	Glücklich
Joyful	Von Freude erfüllt	Freudig erregt
Contented	Zufrieden	Zufrieden
Negative	Negativ	Negativ
Bad	Schlecht	Schlecht
Unpleasant	Unangenehm	Unangenehm
Sad	Traurig	Traurig
Afraid	Ängstlich	Ängstlich
Angry	Wütend	Ärgerlich

Table S3. Results of confirmatory factor analysis (CFA) of the Scale of Positive and Negative Experiences

Model	χ^2	df	CFI	RMSEA [90% CI]	SRMR	AIC
1 Single factor, overall sample	2086.731	54	0.808	0.114 [0.110 - 0.118]	0.074	75128.910
2 Two correlated factors, overall sample	616.156	53	0.947	0.060 [0.056 - 0.065]	0.036	73138.933
3 Two correlated factors, correlated errors, overall sample	315.981	51	0.975	0.042 [0.038 - 0.047]	0.025	72748.867
4 Model 3 as multiple group model for men and women, invariance of factor loadings	555.341	122	0.960	0.049 [0.045 - 0.054]	0.042	72689.605

Overall sample: n = 2,908; multiple group model: n = 1,450 (men), n = 1,458 (women)

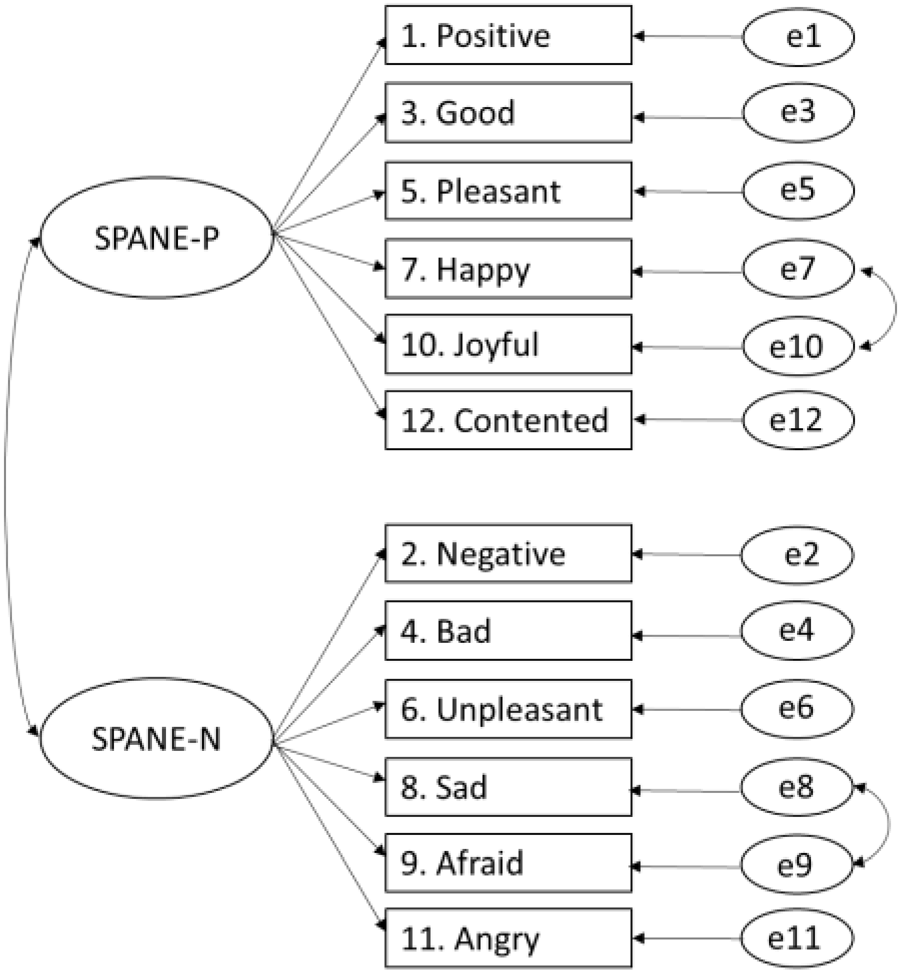

Figure S1. Model 3 of CFA of the Scale of Positive and Negative Experiences (SPANE)

Table S4. Correlations between study variables in men

	2	3	4	5	6	7	8	9	10
1 Vocational training	-0.004	0.037	0.130	0.061	0.186	0.362	-0.135	0.255	-0.203
2 Age	-	0.123	-0.035	-0.051	0.426	-0.349	0.013	-0.100	-0.117
3 Cohabiting partner		-	0.224	0.145	0.157	-0.010	-0.059	0.024	-0.088
4 Life satisfaction			-	0.456	0.148	0.194	-0.141	0.175	-0.248
5 Affect balance				-	0.017	0.108	-0.076	0.089	-0.081
6 Expected income 1999					-	-0.105	-0.149	0.173	-0.447
7 Mean change in income						-	-0.335	0.621	-0.234
8 Current income deviation							-	-0.271	0.215
9 Stability in income development								-	-0.382
10 Share of income from benefits									-

Note: non-parametric correlations, all correlations ≥ |.059| are significant at $p < .05$

Table S5. Correlations between study variables in women.

	2	3	4	5	6	7	8	9	10
1 Vocational training	-0.025	0.004	0.056	0.073	0.113	0.229	0.033	0.050	-0.027
2 Age	-	0.033	-0.057	-0.054	0.260	-0.170	-0.069	0.037	-0.098
3 Cohabiting partner		-	0.240	0.080	-0.053	-0.067	0.010	-0.046	-0.054
4 Life satisfaction			-	0.494	0.008	0.067	-0.012	0.058	-0.139
5 Affect balance				-	-0.009	0.065	0.001	0.029	-0.072
6 Expected income 1999					-	-0.266	-0.043	-0.046	-0.185
7 Mean change in income						-	-0.291	0.597	-0.154
8 Current income deviation							-	-0.220	0.116
9 Stability in income development								-	-0.243
10 Share of income from benefits									-

Note: non-parametric correlations, all correlations ≥ |.050| are significant at $p < .05$

Table S6. Current income deviation (residual 2011) by mean change in income.

	Men		Women	
	Current income deviation		Current income deviation	
**Mean change in income (long-term)**	**Negative[1]**	**No/positive**	**Negative[1]**	**No/positive**
Decrease in income	32%	68%	25%	75%
Increase/no change in income	55%	45%	52%	48%

[1] a negative residual means that the income in 2011 was lower than expected
